# Antisense-mediated splice intervention to treat human disease: the odyssey continues

**DOI:** 10.12688/f1000research.18466.1

**Published:** 2019-05-22

**Authors:** Ianthe Pitout, Loren L. Flynn, Steve D. Wilton, Sue Fletcher

**Affiliations:** 1 Murdoch University, Murdoch, WA, 6150, Australia; 2 The University of Western Australia, Nedlands, WA, 6009, Australia; 3 Perron Institute, Nedlands, WA, 6009, Australia

**Keywords:** antisense oligonucleotide, exon selection, alternative splicing

## Abstract

Recent approvals of oligonucleotide analogue drugs to alter gene expression have been welcomed by patient communities but not universally supported. These compounds represent a class of drugs that are designed to target a specific gene transcript, and they include a number of chemical entities to evoke different antisense mechanisms, depending upon the disease aetiology. To date, oligonucleotide therapeutics that are in the clinic or at advanced stages of translation target rare diseases, posing challenges to clinical trial design, recruitment and evaluation and requiring new evaluation paradigms. This review discusses the currently available and emerging therapeutics that alter exon selection through an effect on pre-mRNA splicing and explores emerging concerns over safety and efficacy. Although modification of synthetic nucleic acids destined for therapeutic application is common practice to protect against nuclease degradation and to influence drug function, such modifications may also confer unexpected physicochemical and biological properties. Negatively charged oligonucleotides have a strong propensity to bind extra- and intra-cellular proteins, whereas those analogues with a neutral backbone show inefficient cellular uptake but excellent safety profiles. In addition, the potential for incorporation of chemically modified nucleic acid monomers, yielded by nuclease degradation of exogenous oligonucleotides, into biomolecules has been raised and the possibility not entirely discounted. We conclude with a commentary on the ongoing efforts to develop novel antisense compounds and enhance oligonucleotide delivery in order to further improve efficacy and accelerate implementation of antisense therapeutics for human disease.

## Introduction

**Figure 1.**
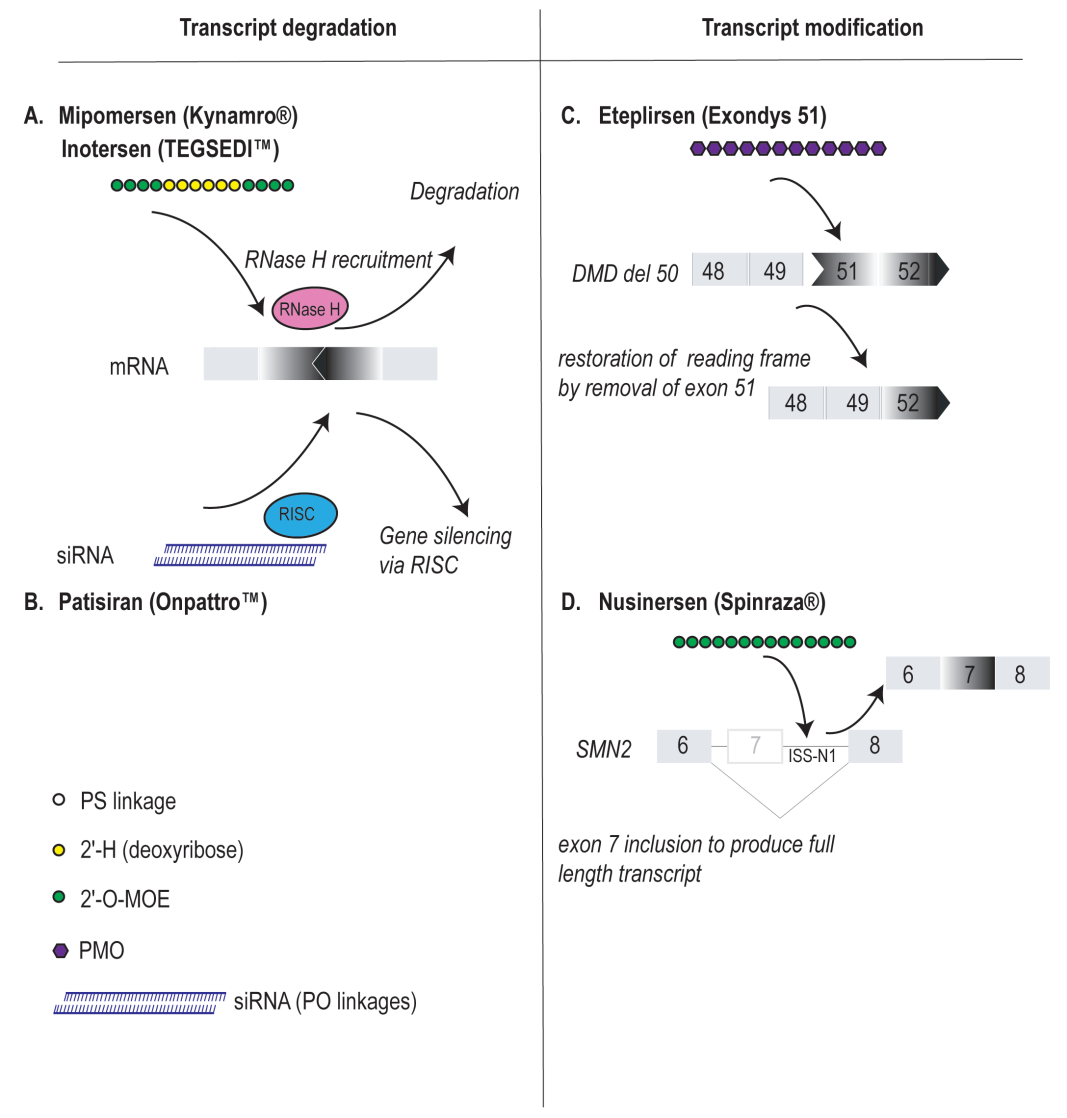
Currently approved antisense oligonucleotide drugs, indicating chemistry and mechanisms of action. Antisense strategies that induce transcript degradation (left panel) include RNaseH degradation of the target transcript, activated by annealing of a complementary oligodeoxynucleotide ( **A**) and transcript degradation by small interfering RNA (siRNA) ( **B**). Transcript modification by RNA analogues (right panel) can be achieved by targeting splice motifs to exclude an exon to alter the reading frame or remove exons carrying mutations ( **C**) or strengthen exon selection, otherwise compromised by base changes ( **D**). 2′-O-MOE, 2′- *O*-(2-methoxyethyl); *DMD*, dystrophin; *ISS-N1*, intron splice silencer N1; PMO, phosphorodiamidate morpholino oligomer; PO, phosphodiester; PS, phosphorothioate; RISC, RNA-induced silencing complex; *SMN2*, survival motor neuron 2.

The integrity of all gene expression is reliant on RNA, and the property of nucleic acids that permits complementary base pairing has long been an attractive option to alter gene expression by modulating RNA structure, function or abundance to treat human disease. Advances in the field have been predicated upon development of synthetic nucleic acid analogues (for review, see 1) that resist nuclease degradation and can be modified to evoke specific mechanisms. An antisense (the reverse complement) nucleic acid sequence (oligonucleotide) to a region of the target molecule can be designed and evaluated in a routine manner to identify compounds with the required specificity and affinity to alter the abundance, structure or functionality of the resulting transcript ( Figure 1). Such outcomes have far-reaching potential to alter the course of human diseases, and at this time, the indications are mostly rare inherited conditions with otherwise limited treatment options. The field has attracted some debate regarding the high treatment costs, clinical trial and regulatory evaluation outcomes and approvals, some uncertainty regarding mechanisms of action ^1^ and emerging reports of off-target effects ^2, 3^. Although only a small number of antisense drugs are in the clinic ^4^ or undergoing translation at this time, ongoing efforts to improve oligonucleotide drug delivery and efficacy and to better understand antisense mechanisms will drive further advances in the field.

**Figure 2.**
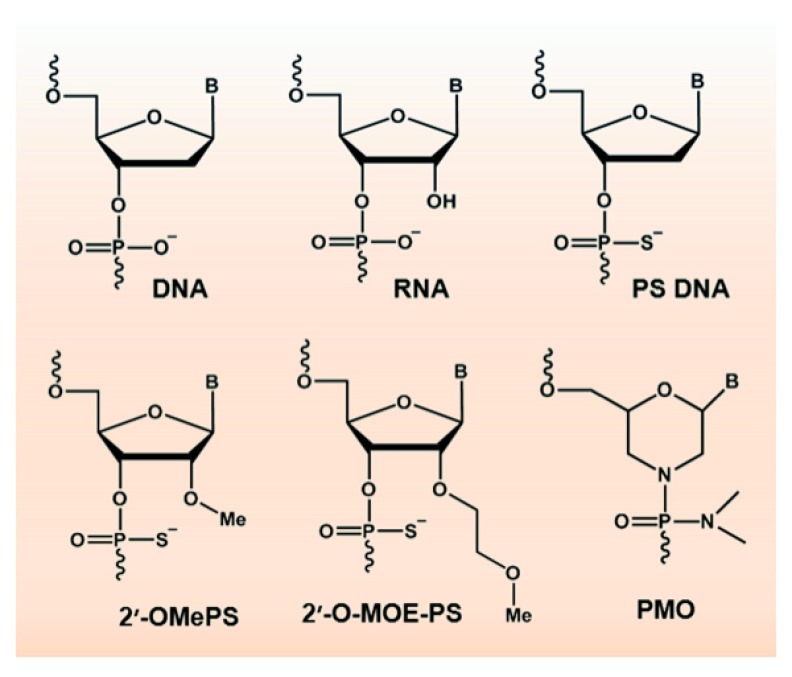
Natural and chemically modified nucleotides. Upper panel: DNA, RNA and phosphorothioate (sulphur substituted for a non-bridging oxygen)-modified nucleotide (PS DNA). Lower panel: 2′- *O*-methyl phosphorothioate (2′-OMePS), 2′- *O*-methoxyethyl phosphorothioate (2′-O-MOE-PS) and phosphorodiamidate morpholino oligomer (PMO).

Natural nucleic acids are biologically labile and their application in drug development has required chemical modifications of the bases and nucleic acid backbone to increase resistance to endogenous nucleases and influence activity. The phosphorothioate backbone is the most widely applied chemical modification ( Figure 2), and although the substitution of sulphur for a non-bridging oxygen confers nuclease stability, changes to the ribose moiety can confer RNA-like characteristics and hydrophobicity to the oligonucleotide (for example, 2′- *O*-methyl, 2′- *O*-methoxyethyl and 2′- *O*-fluoro). Phosphorothioate oligonucleotides carry a strong negative charge, allowing binding to various proteins and complex formation with cationic liposomes for efficient transfection *in vitro.* Other nucleobase and backbone modifications can confer specific characteristics and mechanisms of action, and include peptide nucleic acids and phosphorodiamidate morpholino oligomers (PMOs) ( Figure 2) that carry a neutral charge but show inefficient cellular uptake (for review, see 5).

The more widely exploited antisense strategies use DNA analogues that activate RNase H1 degradation of the target transcript ( Figure 1), and thereby downregulate gene expression, or double-stranded RNAs to induce gene silencing, comprehensively reviewed by Shen and Corey ^6^. The concept of altering splice site selection to modify disease-causing transcripts was validated *in vitro* over 25 years ago ^7^ and requires the design and application of an RNA analogue that targets motifs participating in exon selection and retention in the mature mRNA. Antisense targeting of splice sites or motifs necessary for exon selection can influence endogenous alternative splicing, block aberrant splicing, exclude a cryptic exon or an exon carrying a disease-causing mutation, or restore the open reading frame around a frame-shifting deletion, whereas targeting splicing silencers can enhance selection of an exon, otherwise compromised by nucleotide changes (for reviews, see 8, 9).

At this time, two mRNA splice-modulating drugs are reported to have delivered therapeutic benefit to a subset of patients with Duchenne muscular dystrophy (DMD) ^10^ and to patients with spinal muscular atrophy (SMA) ^11^. *Eteplirsen* ( *Exondys51)*, Sarepta Therapeutics, Cambridge, MA, USA) is an antisense PMO that targets splice enhancer motifs in the *DMD* pre-mRNA to exclude exon 51 and restore the dystrophin mRNA reading frame, disrupted by most deletions beginning at exon 52 or ending at exon 50. *Eteplirsen* received accelerated approval from the US Food and Drug Administration (FDA) in September 2016 but is not yet approved in Europe. The drug *nusinersen* ( *Spinraza* ^®^, Biogen, Cambridge, MA, USA) for the treatment of SMA is a 2′- *O*-methoxyethyl antisense oligonucleotide (AO) on a phosphorothioate backbone. *Nusinersen* targets a splice silencer ( *ISS-N1*) in survival motor neuron 2 ( *SMN2*) intron 7 and promotes exon 7 selection and retention during pre-mRNA splicing ^12^ and was approved by the FDA in December 2016. The roughly 25-year history of antisense drug development parallels that of an increasing interest in therapeutics for rare diseases. Of patients with any of the more than 6,000 rare diseases described to date, only a few groups of patients benefit from an effective treatment. It is perhaps fitting that the first splice intervention antisense therapeutics to reach the clinic address two of the more common and debilitating of the life-limiting inherited rare diseases of childhood. This review focuses on the development and current status of AO drugs that alter exon selection and exert a therapeutic effect by restoring or altering protein structure and function.

## RNA splice-modulating drugs in the clinic

The field of RNA splicing therapeutics was launched by the demonstration of AO-mediated suppression of a disease-causing cryptic splice site in the β-globin transcript in a cell-free system ^7^. This was followed by parallel programs to develop RNA therapeutics to alter exon selection during processing of the dystrophin ( *DMD*) pre-mRNA and generate internally truncated but functional dystrophin isoforms that reflect variants found in the less severe allelic disorder, Becker muscular dystrophy (for review, see 13). Over 60% of the DMD-causing mutations are deletions of one or more exons, predominantly between exons 43 and 53 (major deletion hotspot) or exons 3 and 7 (minor deletion hotspot), that disrupt the translational reading frame and yield a prematurely truncated, non-functional protein. Becker muscular dystrophy is less common and is the consequence of mutations that yield a protein of reduced function or abundance or both. Becker muscular dystrophy mutations are generally in-frame deletions and provide “templates” for partially functional dystrophin variants that could ameliorate the severe dystrophic muscle phenotype characteristic of DMD. While the dystrophin exon-skipping programs aimed to develop antisense oligomer sequences that prevent exon selection during dystrophin pre-mRNA splicing (exon skipping) to generate internally truncated dystrophin molecules, two different RNA-like analogue chemistries were used for translational development.

### Antisense treatment to restore dystrophin expression

Two experimental antisense drugs, both designed to treat DMD in a subset of patients by removing dystrophin exon 51 and re-frame the transcript around disease-causing deletions that flank exon 51, such as exon 50, exons 48 to 50 or exon 52, were evaluated in independent clinical trials and presented for regulatory consideration in 2015. The investigational drug *drisapersen*, a 2′- *O*-methyl–modified AO on a phosphorothioate backbone, was evaluated in 186 ambulant participants with DMD by using subcutaneous injections at a dosage of 6 mg/kg per week in an extended study over the course of 188 weeks ^14^. Although the drug was reported to be generally well tolerated ^14^, renal effects, thrombocytopenia and injection site reactions have attracted attention ^15^. *Drisapersen* failed to meet primary and secondary endpoints and, in light of the considerable adverse side effects, was withdrawn from further development after negative feedback from the regulators. (For comprehensive review of antisense therapeutics for neuromuscular disease, see 16.) Injection site reactions and skin abnormalities as a consequence of long-term subcutaneous *drisapersen* injection remain unresolved even long after cessation of treatment ^15, 17^.

*Eteplirsen* is a PMO delivered by once-weekly intravenous infusion (30 mg/kg) that also targets dystrophin exon 51 and received accelerated approval from the FDA in 2016. The approval of *eteplirsen* evoked lively discussion ^18– 21^ largely because of the small study group; use of a trial structure that had the placebo cohort transition to treatment after 6 months, when the difference in ambulation, the primary endpoint, reached significance; and the low (relative to healthy) levels of muscle dystrophin induced by the treatment ^10^. In the aftermath of FDA review and concern regarding the efficacy of *eteplirsen*, much attention focused on dystrophin quantitation and clinical trial outcome measures. However, seven years after initiation of Sarepta Therapeutics study 201 and completion of the extension study 202 (long-term safety and efficacy), the trial participants receiving *eteplirsen* continue to show functional benefits, respiratory function decline is half that expected from the natural history of the disease ^22^, and no treatment-related serious adverse events have been reported. Class II evidence of increased dystrophin expression over baseline dystrophin was presented ^23^, and although the treatment regimen is costly (more than $300,000 per year for a 25-kg child; for review, see 24) and the weekly intravenous treatment regimen can be burdensome for patients and their families, continued ambulation in teenage patients with DMD ^10^, together with respiratory benefits ^22^, represents a marked deviation from the natural history of the disease, is unprecedented and sets a benchmark for future dystrophin-restoring therapies.

### Spinal muscular atrophy

SMA is a recessive disease characterized by deficiency of SMN protein that leads to the selective loss of spinal motor neurons and skeletal muscle paralysis, affecting the trunk and limbs. SMA is the most common genetic cause of infant mortality with a prevalence of 1 in about 11,000 live births and carrier frequency of 1 in 40 to 67, depending on ethnicity ^25^. The genetic defect in 95% of cases is homozygous deletion of the *SMN1* gene and while the *SMN2* gene, present in all patients with SMA, potentially encodes an identical protein, a synonymous C>T variant in exon 7 leads to aberrant processing of the mRNA. *SMN2* copy number is polymorphic, and there is some inverse correlation between the number of copies of *SMN2* and disease severity. An AO targeting an intronic splice silencer motif, *ISS-N1* ^12^, increased full-length *SMN* transcript in pre-clinical studies, and the 2′- *O*-methoxyethyl phosphorothioate AO was approved under the name *spinraza* after a randomized placebo-controlled clinical trial *.* The patients who received intrathecal injection of *nusinersen* (12 mg) showed increased event-free survival and significant improvements in motor function, not seen in patients with SMA type 1 ^11^, prompting early termination of the study ^26^. Comprehensive review and analysis of *nusinersen* treatment in SMA by the Canadian Agency for Drugs and Technologies in Health include commentary on the cost-benefit (at a cost of $708,000 in year 1 for four treatments of 12 mg and $354,000 annually for maintenance treatments thereafter) ^27^. *Nusinersen* is now available in many European countries, the USA, Canada, Australia, New Zealand and Japan and is reported to deliver clinical benefit in both SMA type 1 and type 2 patients ^28^.

## Splice-modifying therapies under development

Pre-clinical and clinical studies on novel splice-modulating experimental drugs are gaining momentum. (For review of splice-modifying oligonucleotides under development, see 9.) Current strategies include dystrophin exon-skipping programs, exons 45 *casimersen* (SRP-4045) and 53 *golodirsen* (SRP-4053) by Sarepta Therapeutics ( https://www.sarepta.com/pipeline/exon-skipping-duchenne); an unrelated dystrophin exon 53 program ^29, 30^; exon-skipping strategies to treat dysferlinopathies (for example, limb girdle muscular dystrophy type 2B) by excluding exons 37 and 38 in a *Dysf* mutant mouse model; targeting cryptic exons activated by deep intronic mutations causing choroideremia ^31^, Leber congenital amaurosis ^32^ and USH2A-associated retinal degeneration ^33^; correction of mis-splicing of harmonium in USH1C and exon inclusion to address a common splice variant causing adult-onset Pompe disease ^34^. Two different exon-skipping strategies to treat spinocerebellar ataxia type 3 were recently reported. The pathogenic expanded polyglutamine repeat can be removed by skipping exon 10 ^35^; however, in an innovative strategy, exons 8 and 9 were skipped in order to prevent proteolytic cleavage and generation of toxic protein fragments ^36^.

Though not widely exploited, modulation of endogenous alternative splicing to alter isoform ratios could have broad applicability to a number of acquired and inherited phenotypes. AO-mediated switching of *Bcl-x* splicing from the anti-apoptotic isoform to the pro-apoptotic isoform induced apoptosis of glioma cell lines ^37^ and of hepatic stellate cells in liver fibrosis studies ^38^. Endogenous aberrant splicing of *GNB3* was corrected by AO-mediated splice intervention ^39^. The *LMNA* gene encodes two alternatively spliced products that are major components of the nuclear envelope. Mutations in the pre-lamin A transcript can result in aberrant splicing, causing Hutchison–Gilford progeria syndrome that can be addressed either by AO targeting of the cryptic splice site (for review, see 40) or by manipulating splicing of exon 11 to favour the lamin C isoform ^41^.

Although development of novel molecular therapeutics is eagerly anticipated by patients and families and has instigated efforts to find new targets and intervention opportunities, current limitations to broader applicability need to be overcome, primarily inefficient delivery to target tissues. The charge-neutral PMOs have an excellent safety profile but are rapidly cleared from the circulation, showing poor uptake into cells. Pre-clinical development of exon-skipping PMOs in a mouse muscular dystrophy model showed moderate restoration of skeletal muscle dystrophin but minimal uptake in the myocardium ^42^. Systemic delivery of these molecules can be greatly improved by conjugation to cell-penetrating peptides ^43, 44^, the use of novel strategies, such as exploiting exosomes and targeting peptides ^45^, and gaining greater insights into the mechanisms of cell uptake ^46^. Successful translation of peptide-phosphorodiamidate morpholino drugs that are currently in development and clinical evaluation (for example, for DMD) may well begin a new era for splice-modifying therapies, particularly if tissue or cell targeting can be achieved, perhaps leading to reduced dosages.

## Non-antisense and off-target effects of synthetic oligonucleotides

Oligonucleotide backbone and sugar modifications influence resistance to nucleases, mechanism of action, tissue distribution and binding to various proteins but can also cause hybridization-dependent (off-target hybridization of RNase H1-competent AOs resulting in hepatotoxicity ^47^) and independent effects. Phosphorothioate oligonucleotides administered *in vivo* bind to serum and cellular proteins ^2, 48^ (for review, see 49) and show good tissue uptake and distribution, in the brain in particular ^50^. However, in a systematic literature review of original trial reports, by van Meer *et al*. ^51^, all studies involving phosphorothioate oligonucleotides administered subcutaneously report injection site reactions, while intravenous delivery was associated with thrombocytopenia. Phosphorothioate oligonucleotide sequences were reported to be immunostimulatory ^52^ and to bind to platelets, eliciting strong platelet-activating effects ^53^, while intracerebroventricular injection of 2′- *O*-methyl phosphorothioate AOs upregulated immune system-associated genes ^50^. More recently, antisense drugs include modifications to enhance affinity (for example, locked nucleic acids or constrained ethyl substituted gapmers) that are reported to cause hepatotoxicity due to off-target RNA hybridization and downregulation ^47, 54^. In an elegant study, Dieckmann *et al*. ^54^ showed that hybridization-dependent toxicity of RNase H1-competent phosphorothioate oligonucleotides can be mitigated by limiting the affinity of the oligonucleotide and improving target specificity.

The propensity for phosphorothioate oligonucleotides to cause injection site reactions ^15, 17^, flu-like symptoms, and hepatotoxicity was well known prior to the *drisapersen* exon-skipping studies and was reported in patients who received the approved drug *mipomersen* (RNase H1-competent gapmer) for the treatment of homozygous hypercholesteremia ^55, 56^.

Although the hepatotoxicity may be attributable in part to off-target hybridization, the exact cause of the injection site reactions remains unknown. Shen *et al*. ^57^ examined cells transfected with phosphorothioate oligonucleotides with various 2′ modifications. They found that these compounds bind a large number of intracellular proteins and can replace the architectural long non-coding RNA *NEAT1* in subnuclear bodies called paraspeckles. The repertoire of proteins bound by phosphorothioate AOs and the effects of protein depletion or altered cellular localization are influenced by any 2′ modification ^58^. The most widely used modification, 2′- *O*-methoxyethyl, was found to have an acceptable tolerability profile and consistent behaviour between sequences ^3^, whereas 2′-fluoro-modified phosphorothioate oligonucleotides disturb nuclear biology, impair cell proliferation and cause loss of cellular proteins ^59^.

We explored the interaction of 2′- *O*-methyl phosphorothioate AOs with nuclear proteins and report backbone-specific effects of modified oligonucleotides on subnuclear organelles, altered distribution of nuclear proteins, the appearance of novel large structured nuclear inclusions, modification of RNA processing and marked disturbance of the transcriptome in cultured cells. We speculate that some side effects and adverse events reported after clinical evaluation of phosphorothioate nucleic acid drugs may be mediated, at least in part, by non-specific interaction of nuclear components with the phosphorothioate backbone ^60^. The sequence-independent effects of phosphorothioate compounds indeed may enhance or confound apparent antisense effects ^61^ as a consequence of extensive disturbance of nuclear proteins and organelles, particularly those critical for RNA processing and splicing. Strategies to reduce cellular protein binding by phosphorothioate AOs and mitigate toxic effects, thereby improving the therapeutic profile of these molecules by backbone ^62^ and 2′- *O*-modifications ^63^ are important steps in guiding AO design. Other considerations that may warrant comprehensive investigation are whether phosphorothioate nucleotide degradation products are incorporated into cellular nucleic acids, a question that has been raised ^64^, and whether the immune activation by intra-cerebroventricular injection of a 2′- *O*-methyl phosphorothioate AO reported in mice ^50^ should be of concern in humans who receive similar compounds.

## Molecular medicines in the future

In an era of promising gene replacement and gene correction strategies, exploiting novel recombinant viruses that can be selected and tailored for tropism to specific tissues, the rationale for pursuing transcript-modifying therapies might be questioned. Many of these emerging molecular and gene therapeutics target rare and life-limiting diseases caused by multiple gene variants that may show variable progression and phenotypes within one patient group and, in the event that more than one treatment modality is investigated, compete for the same restricted patient population in recruiting clinical trial participants.

However, consider the situation where different treatment modalities could be used in tandem. For example, an antisense strategy might be the only option following vector gene delivery if the virus results in seroconversion and precludes re-delivery. PMOs designed to alter exon selection are specific to the target transcript and, to the best of our knowledge, have not been reported to cause off-target effects on splicing and have no effect when delivered to cells lacking the target transcript. In the case of cryptic exon or aberrant splice site usage, antisense-mediated strategies can generate a normal product, unlikely to be immunogenic, that is expressed under endogenous, tissue/cell/developmental regulation. Gain-of-function mutations may be particularly amenable to splice interventions, whereas for loss-of-function mutations, splice variants can be designed to encode products that may exhibit reduced function but could ameliorate otherwise severe disease.

We conclude by acknowledging the contribution of the patients and the combined efforts of chemists, researchers and clinicians and those in the biopharmaceutical industry who championed these drugs and the insightful regulators who have yielded two splice interventions, now available to patients. Although there is general agreement that better drug uptake and greater efficacy are desirable, these drugs, by all accounts, are altering the course of previously untreatable disease. There is potential for numerous therapeutics in the “splice modulating” class. The lessons learnt over the course of translating *eteplirsen* and *nusinersen* and from the failure of *drispersen* will inform the development of emerging and future molecular medicines.

## Abbreviations

AO, antisense oligonucleotide; DMD, Duchenne muscular dystrophy; FDA, US Food and Drug Administration; PMO, phosphorodiamidate morpholino oligomer; SMA, spinal muscular atrophy; SMN, survival motor neuron
